# A model for the Twitter sentiment curve

**DOI:** 10.1371/journal.pone.0249634

**Published:** 2021-04-15

**Authors:** Giacomo Aletti, Irene Crimaldi, Fabio Saracco

**Affiliations:** 1 ADAMSS Center, Università degli Studi di Milano, Milan, Italy; 2 IMT School for Advanced Studies, Lucca, Italy; Lingnan University, HONG KONG

## Abstract

Twitter is among the most used online platforms for the political communications, due to the concision of its messages (which is particularly suitable for political slogans) and the quick diffusion of messages. Especially when the argument stimulate the emotionality of users, the content on Twitter is shared with extreme speed and thus studying the tweet sentiment if of utmost importance to predict the evolution of the discussions and the register of the relative narratives. In this article, we present a model able to reproduce the dynamics of the sentiments of tweets related to specific topics and periods and to provide a prediction of the sentiment of the future posts based on the observed past. The model is a recent variant of the Pólya urn, introduced and studied in Aletti and Crimaldi (2019, 2020), which is characterized by a “local” reinforcement, i.e. a reinforcement mechanism mainly based on the most recent observations, and by a random persistent fluctuation of the predictive mean. In particular, this latter feature is capable of capturing the trend fluctuations in the sentiment curve. While the proposed model is extremely general and may be also employed in other contexts, it has been tested on several Twitter data sets and demonstrated greater performances compared to the standard Pólya urn model. Moreover, the different performances on different data sets highlight different emotional sensitivities respect to a public event.

## 1 Introduction

In the last few years, the internet has become the main source for news for citizens both in EU [1] and in USA [2]. Such a rapid change in the media system has created a symmetric change in the way news are delivered: before the diffusion of the web, information was *intermediated* by journals, newspapers, radio and TV newscast, that represented the *authority*, being publicly responsible for the diffusion of reliable news. Nowadays, such intermediation is not present anymore: every blog or account on Facebook or Twitter assumes truthfulness just for existing online [3–6]. Due to this abrupt change of paradigm in the fruition of news, we observe a great increase of the diffusion of misinformation [7–9], that appears on the web via the use of automated [10–16] or genuine accounts [4, 16–20]. It has been observed that the diffusion of disinformation or misinformation campaigns leans on the emotionality of users [3, 4, 6, 21].

Twitter is one of the most famous microblogging service, where people freely express their views and feelings in short messages, called tweets [22]. Twitter is reknown to be used especially for the political communications [23], due to the limited amount of characters, perfectly suitable for political slogans, and for the quick sharing of messages. Due to the availability of its data, via the official API, it represents an extremely rich resource of “spontaneous emotional information” [24]. Sentiment analysis, also known as opinion mining, is a collection of techniques in order to automatically detect the positive or negative connotation of texts. An overview of the latest tools, updates and open issues in sentiment analysis can be found in [25–27] (see also the references therein). Some examples of applications, where predictions are formulated based on the sentiment extracted from on-line texts are provided in [28–35]. In [36], sentiment analysis is used to investigate the emotion transmission in e-communities; while in [37], it is employed in order to investigate on the interplay between macroscopic socio-economic, political or cultural events and the public mood trends, showing that these events have a significant and immediate effect on various aspects of public mood. The Ref. [24] provides a matrix-factorization method to predict individuals’ opinions toward specific topics they had not directly given. In [38], the authors consider the sentiment curve of Twitter posts along time in order to infer the causes of sentiment variations, leveraging on the idea that the emerging topics discussed in the variation period could be highly related to the reasons behind the variations. In [39], the authors present the data prediction as a process based on two different levels of granularity: i) a fine-grained analysis to make tweet-level predictions on various aspects, such as sentiment, topics, volume, location, time-frame, and ii) a coarse-grained analysis to predict the outcome of a real-world event, by aggregating and combining the fine-grained predictions. With respect to this classification, the present work can be placed in the stream of literature regarding the fine-grained analysis to model/predict the sentiment of Twitter posts.

While an important body of research target the issue of predicting the information cascades [40–47], to the best of our knowledge, there are not works that provide models for the evolution of Twitter sentiment. We aim at filling in this gap, presenting a model that is able to reproduce the sentiment curve of the tweets related to specific topics and periods and to provide a prediction of the sentiment of the future posts based on the observed past. We achieve this purpose employing a recent variant of the Pólya urn, introduced in [48] and called Rescaled Pólya (RP) urn. In brief, the RP urn model differs from the standard Pólya urn for the presence of a “local” reinforcement, i.e. elements that are recently observed have a greater impact on the near future and may be identified as the “fashion” of the moment. In the online social networks applications, this local reinforcement aims at representing the persistence of an emotional response to a public event, capturing the phenomenon observed in [3]. Moreover, it is able to correctly reproduce the sentiment dynamics of the tweets, outperforming the standard Pólya urn model, as we will see, on several different data sets. Its prediction ability is also quite high. It is important to note that we also include a delay in information: indeed, it is plausible that, when the user decides to write the tweet posted at time-step *n* + 1, he /she only knows the previous tweets until a certain time-step *t*(*n*)<*n*.

Finally, we underline that the proposed model may be also employed in other contexts.

The sequel of the work is so structured. In Section 2 we will present the model: after introducing the standard Pólya model in Subsection 2.1, in Subsection 2.2 we formally describe the Rescaled Pólya urn model in general and, then, we focus on the case with two colors and, next to the general model (Complete model), we identify two special cases (“Only fashion” model and “No fashion” model). Finally, in Subsection 2.3, we explain how we include a delay in information. In Section 3, we describe the considered datasets and we illustrate the performed analysis and the obtained results. In Section 4 we comment the results and draw our conclusions. The paper is enriched by an appendix regarding the evolution of the estimated model parameters and additional analyses.

## 2 Model

### 2.1 Standard Pólya urn

The standard Pólya urn (see [49–51]) is a stochastic model driven by a reinforcement mechanism (also known as “rich get richer” principle): the probability that a given event occurs increases with the number of times the same event occurred in the past. This rule is a key feature governing the dynamics of many biological, economic and social systems (see, e.g. [51]) and it seems plausible that it plays a role also in the sentiment dynamics of the Twitter posts as the emotional state of an individual influences the emotions of others [36, 52]. The Pólya urn model has been widely studied and generalized (some recent variants can be found in [48, 53–65]) and in its simplest form, with *c*-colors, works as follows. An urn contains *N*_0*i*_ balls of color *i*, for *i* = 1, …, *c*, and, at each discrete time-step, a ball is extracted from the urn and then it is returned inside the urn together with *α* > 0 additional balls of the same color. Therefore, if we denote by *N*_*ni*_ the number of balls of color *i* in the urn at time-step *n*, we have

$$\begin{array}{c} N_{n\;i}=N_{n-1\;i}+\alpha \xi_{n\;i}=N_{0\;i}+\alpha \sum_{h=1}^{n}\xi_{h\;i}\;\text{for}\;n\geq 1, \end{array}$$

where *ξ*_*ni*_ = 1 if the extracted ball at time-step *n* is of color *i*, and *ξ*_*ni*_ = 0 otherwise. The parameter *α* regulates the reinforcement mechanism: the greater *α*, the greater the dependence of *N*_*ni*_ on $\sum_{h=1}^{n}\xi_{h\;i}$.

### 2.2 Rescaled Pólya (RP) urn

The “Rescaled” Pólya (RP) urn model, introduced in [48], is characterized by the introduction of the parameter *β*, together with the initial parameters (*b*_0*i*_)_*i* = 1, …, *c*_ and (*B*_0*i*_)_*i* = 1, …, *c*_, next to the parameter *α* of the original model, so that

$$\begin{array}{c} \begin{array}{cccc} N_{n\;i} & =b_{0\;i}+B_{n\;i} & & \text{with}\;\\ B_{n\;i} & =\beta B_{n-1\;i}+\alpha \xi_{n\;i} & & n\geq 1. \end{array} \end{array}$$

**Fig 1:**
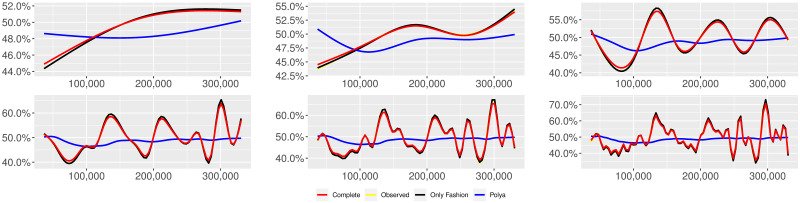
“Migration” (*T* = 0.35, entire, *D* = 3′, *S* = 100 slots of equal size): Sentiment curves. In each panel, the yellow line is the cubic spline smoothing of the time series of the observed tweets *ξ*_*n*+1_, together with the default confidence interval (gray), the red line represents the cubic spline smoothing of the time series of the estimated predictive means ${\hat{\psi }}_{n}$ (defined in Subsec. 3.2), obtained with the complete RP model, the black and the blue lines provide similar approximations obtained with the other models: black = Only fashion RP model and blue = Standard Pólya model. In each panel, the smoothing is obtained with a given number of nodes: *k* = 3 (top left panel), 5 (top middle panel), 10 (top right panel), 20 (bottom left panel), 30 (bottom middle panel), 50 (bottom right panel).

**Fig 2:**
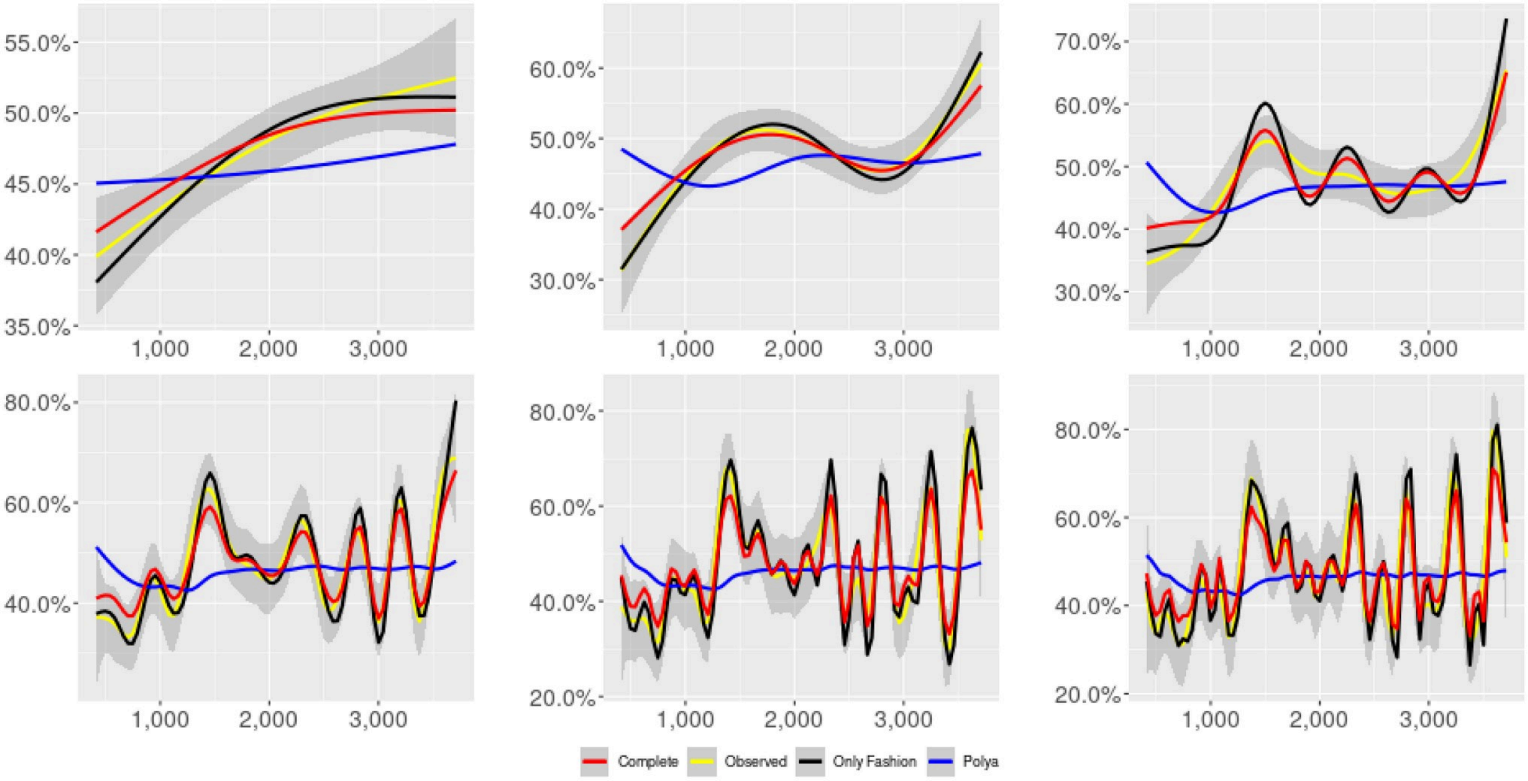
“Migration” (*T* = 0.35, only BOTs’ posts, *D* = 3′, *S* = 100 slots of equal size): Sentiment curves for BOTs’ posts. In each panel, the yellow line is the cubic spline smoothing of the time series of the observed tweets *ξ*_*n*+1_, together with the default confidence interval (gray), the red line represents the cubic spline smoothing of the time series of the estimated predictive means ${\hat{\psi }}_{n}$ (defined in Subsec. 3.2), obtained with the complete RP model, the black and the blue lines provide similar approximations obtained with the other models: black = Only fashion RP model and blue = Standard Pólya model. In each panel, the smoothing is obtained with a given number of nodes: *k* = 3 (top left panel), 5 (top middle panel), 10 (top right panel), 20 (bottom left panel), 30 (bottom middle panel), 50 (bottom right panel).

**Fig 3:**
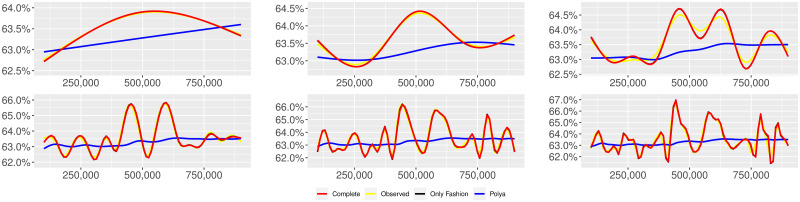
“10 days of traffic” (*T* = 0.35, entire, *D* = 30′′, *S* = 100 slots of equal size): Sentiment curves. In each panel, the yellow line is the cubic spline smoothing of the time series of the observed tweets *ξ*_*n*+1_, together with the default confidence interval (gray), the red line represents the cubic spline smoothing of the time series of the estimated predictive means ${\hat{\psi }}_{n}$ (defined in Subsec. 3.2), obtained with the complete RP model, the black and the blue lines provide similar approximations obtained with the other models: black = Only fashion RP model and blue = Standard Pólya model. In each panel, the smoothing is obtained with a given number of nodes: *k* = 3 (top left panel), 5 (top middle panel), 10 (top right panel), 20 (bottom left panel), 30 (bottom middle panel), 50 (bottom right panel).

**Fig 4:**
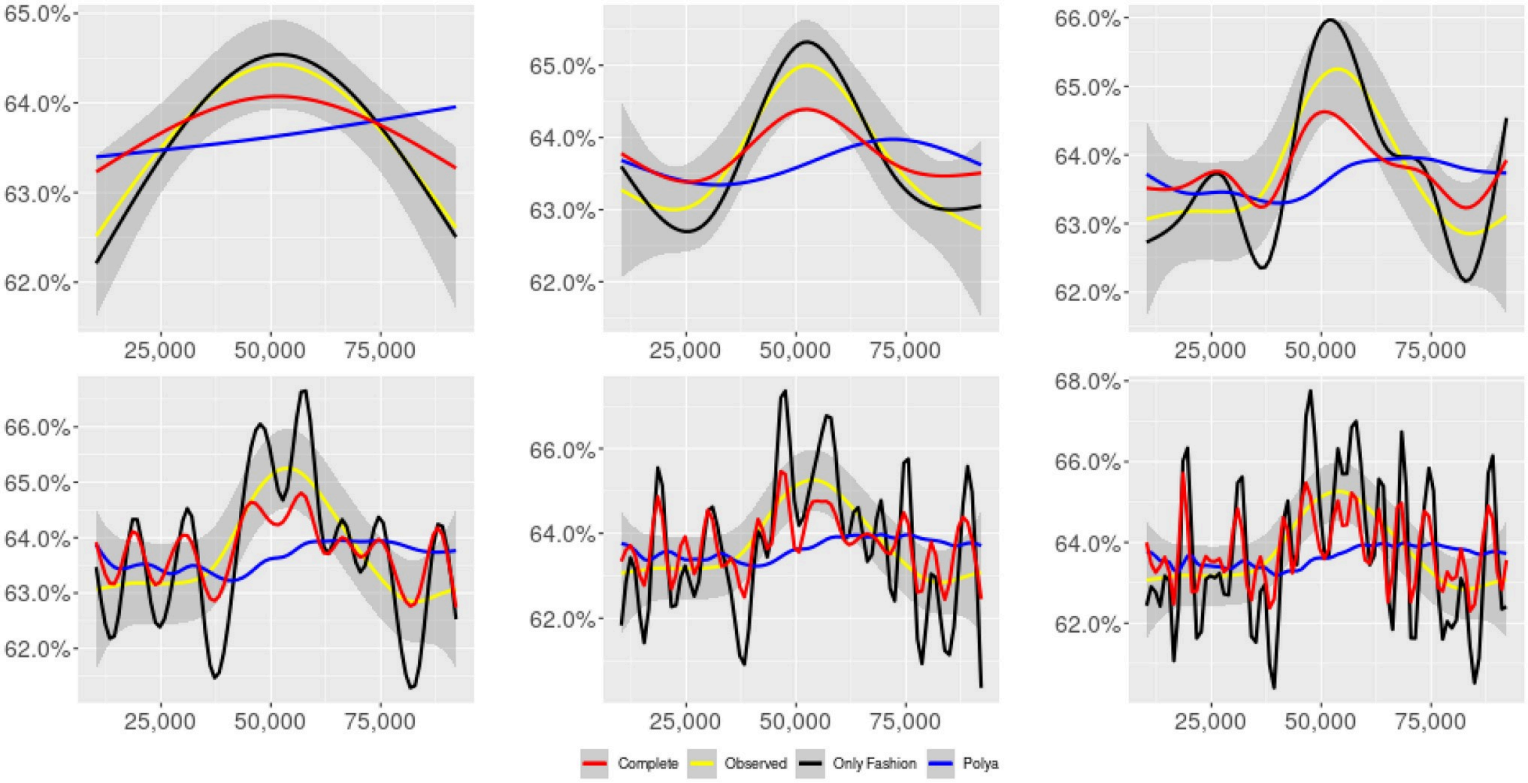
“10 days of traffic” (*T* = 0.35, only BOTs’ posts, *D* = 30′′, *S* = 100 slots of equal size): Sentiment curves for BOTs’ postsx. In each panel, the yellow line is the cubic spline smoothing of the time series of the observed tweets *ξ*_*n*+1_, together with the default confidence interval (gray), the red line represents the cubic spline smoothing of the time series of the estimated predictive means ${\hat{\psi }}_{n}$ (defined in Subsec. 3.2), obtained with the complete RP model, the black and the blue lines provide similar approximations obtained with the other models: black = Only fashion RP model and blue = Standard Pólya model. In each panel, the smoothing is obtained with a given number of nodes: *k* = 3 (top left panel), 5 (top middle panel), 10 (top right panel), 20 (bottom left panel), 30 (bottom middle panel), 50 (bottom right panel).

**Fig 5:**
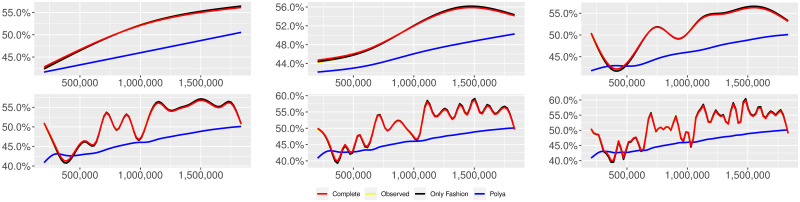
“Covid” (*T* = 0.35, entire, *D* = 3′, *S* = 1000 slots of equal size): Sentiment curves. In each panel, the yellow line is the cubic spline smoothing of the time series of the observed tweets *ξ*_*n*+1_, together with the default confidence interval (gray), the red line represents the cubic spline smoothing of the time series of the estimated predictive means ${\hat{\psi }}_{n}$ (defined in Subsec. 3.2), obtained with the complete RP model, the black and the blue lines provide similar approximations obtained with the other models: black = Only fashion RP model and blue = Standard Pólya model. In each panel, the smoothing is obtained with a given number of nodes: *k* = 3 (top left panel), 5 (top middle panel), 10 (top right panel), 20 (bottom left panel), 30 (bottom middle panel), 50 (bottom right panel).

**Fig 6:**
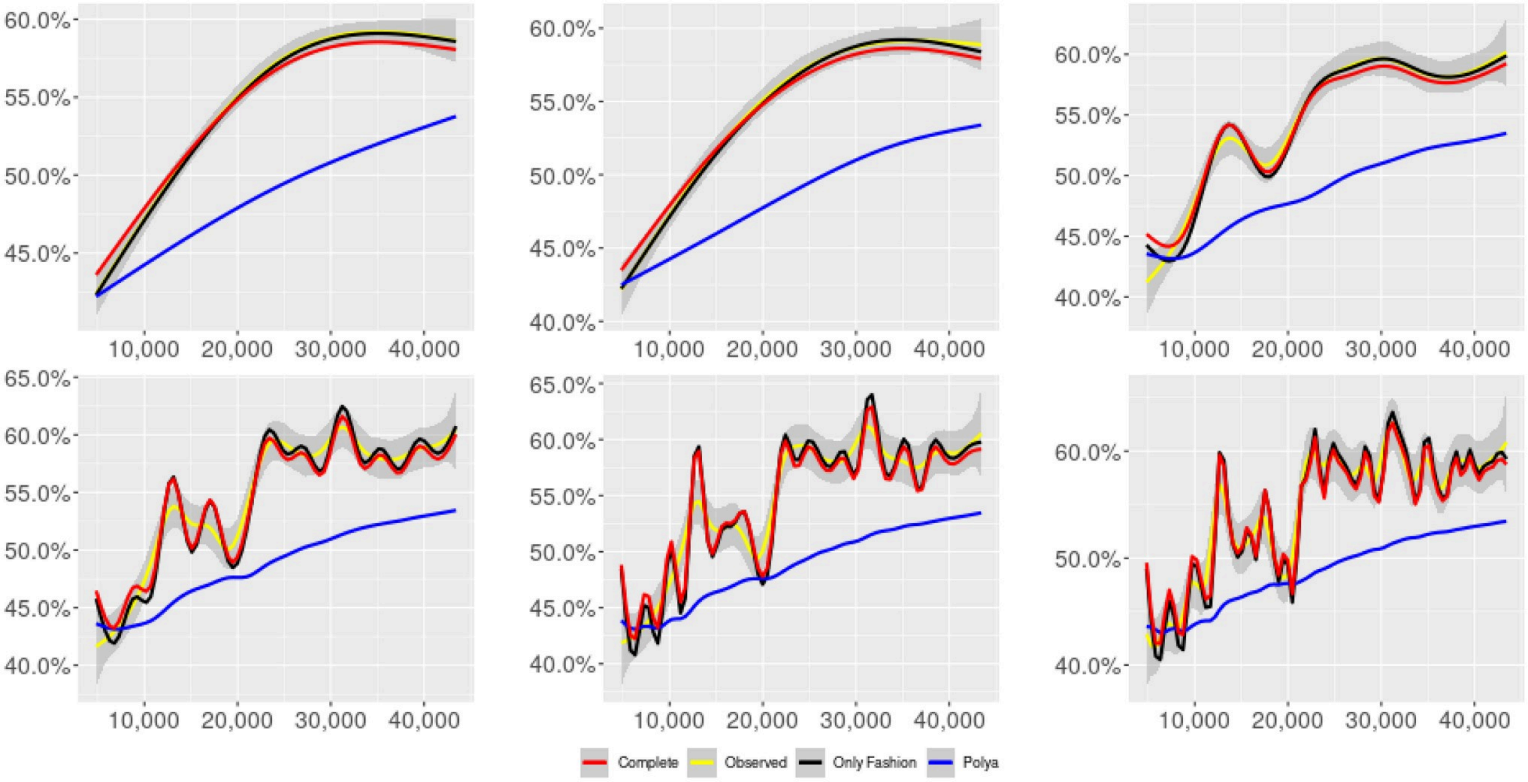
“Covid” (*T* = 0.35, only BOTs’ posts, *D*′ = 3, *S* = 1000 slots of equal size): Sentiment curves for BOTs’ posts. In each panel, the yellow line is the cubic spline smoothing of the time series of the observed tweets *ξ*_*n*+1_, together with the default confidence interval (gray), the red line represents the cubic spline smoothing of the time series of the estimated predictive means ${\hat{\psi }}_{n}$ (defined in Subsec. 3.2), obtained with the complete RP model, the black and the blue lines provide similar approximations obtained with the other models: black = Only fashion RP model and blue = Standard Pólya model. In each panel, the smoothing is obtained with a given number of nodes: *k* = 3 (top left panel), 5 (top middle panel), 10 (top right panel), 20 (bottom left panel), 30 (bottom middle panel), 50 (bottom right panel).

Therefore, the urn initially contains *b*_0*i*_ + *B*_0*i*_ > 0 balls of color *i* and the parameter *β* ≥ 0, together with *α* > 0, regulates the reinforcement mechanism. More precisely, the term *βB*_*n*−1*i*_ links *N*_*ni*_ to the “configuration” at time-step *n* − 1 through the “scaling” parameter *β*, and the term *αξ*_*ni*_ links *N*_*ni*_ to the outcome of the extraction at time-step *n* through the parameter *α*. Note that the case *β* = 1 corresponds to the standard Pólya urn with an initial number *N*_0*i*_ = *b*_0*i*_ + *B*_0*i*_ of balls of color *i*. When *β* ∈ [0, 1), this variant of the Pólya urn is characterized by a “local” reinforcement, i.e. a reinforcement mechanism mainly based on the most recent observations, and by a random persistent fluctuation of the predictive mean *ψ*_*ni*_ = *E*[*ξ*_*n*+1*i*_ = 1 |“past”]. As we will show, this latter feature is capable of capturing the trend fluctuations in the sentiment curve of Twitter posts (see Figs 1–6).

More formally, given a vector $x={\left(x_{1},\ldots ,x_{c}\right)}^{⊤}\in R^{c}$, we define $\left|x\right|=\sum_{i=1}^{c}\left|x_{i}\right|$. Moreover, we set ***b***_**0**_ = (*b*_01_, …, *b*_0*c*_)^⊤^ and ***B***_**0**_ = (*B*_01_, …, *B*_0*c*_)^⊤^, we assume |***b***_**0**_|>0 and we define $p_{0}=\frac{b_{0}}{|b_{0}|}$. At each discrete time-step (*n* + 1)≥1, a ball is drawn at random from the urn, obtaining the random vector ***ξ***_***n***+**1**_ = (*ξ*_*n*+11_, …, *ξ*_*n*+1*c*_)^⊤^ defined as

$$\begin{array}{c} \xi_{n+1\;i}=\{\begin{array}{cc} 1 & \text{when}\;\text{the}\;\text{extracted}\;\text{ball}\;\text{at}\;\text{time-step}\;n+1\;\text{is}\;\text{of}\;\text{color}\;i\\ 0 & \text{otherwise}\;. \end{array} \end{array}$$

The number of balls in the urn is so updated:

$$\begin{array}{c} N_{n+1}=b_{0}+B_{n+1}\;\text{with}\;B_{n+1}=\beta B_{n}+\alpha \xi_{n+1}\;, \end{array}$$ (1)

which gives (since |***ξ***_***n***+**1**_| = 1)

$$\begin{array}{c} |B_{n+1}\left|=\beta \right|B_{n}|+\alpha . \end{array}$$ (2)

Therefore, setting $r_{n}^{*}=|N_{n}\left|=\right|b_{0}\left|+\right|B_{n}|$, we get

$$\begin{array}{c} r_{n+1}^{*}=r_{n}^{*}+\left(\beta -1\right)\left|B_{n}\right|+\alpha . \end{array}$$ (3)

Moreover, denoting by $F={\left(F_{n}\right)}_{n\geq 0}$ the filtration representing the information along time-steps (formally, this means to set $F_{0}$ equal to the trivial *σ*-field and $F_{n}=\sigma \left(\xi_{1},\ldots ,\xi_{n}\right)$ for *n* ≥ 1), the conditional probabilities ***ψ***_***n***_ = (*ψ*_*n*1_, …, *ψ*_*nc*_)^⊤^ of the extraction process, also called predictive means, are

$$\begin{array}{c} \psi_{n\;i}=E\left[\xi_{n+1\;i}\;|\;F_{n}\right]=P\left(\xi_{n+1\;i}=1|F_{n}\right)=\frac{N_{n\;i}}{|N_{n}|}=\frac{b_{0\;i}+B_{n\;i}}{r_{n}^{*}},\;i=1,\ldots c,\;n\geq 0\;. \end{array}$$ (4)

This urn model has been studied in [48, 53]. All the mathematical proofs and details can be found in these papers.

#### 2.2.1 Two colors (*c* = 2)

With two colors, the quantity of interest are only *ξ*_*n*_ = *ξ*_*n*1_ = 1 − *ξ*_*n*2_ and *ψ*_*n*_ = *ψ*_*n*1_ = 1 − *ψ*_*n*2_. In the sequel, we consider the RP urn model with *β* = 1 (i.e. the standard Pólya urn model) and with *β* < 1. In the first case, we have

$$\begin{array}{c} \psi_{n}=\frac{N_{0\;1}+\alpha \sum_{h=1}^{n}\xi_{h}}{|N_{0}|+\alpha n}\;. \end{array}$$

In the second case, by (1), (2), (3) and (4), using $\sum_{m=0}^{n-1}x^{m}=\left(1-x^{n}\right)/\left(1-x\right)$, we obtain

$$\begin{array}{c} r_{n}^{*}=|b_{0}|+\frac{\alpha }{1-\beta }+\beta^{n}(|B_{0}|-\frac{\alpha }{1-\beta })⟶r^{*}=\left|b_{0}\right|+\frac{\alpha }{1-\beta } \end{array}$$

and

$$\begin{array}{c} \psi_{n}=\frac{b_{0\;1}+\beta^{n}B_{0\;1}+\alpha \sum_{h=1}^{n}\beta^{n-h}\xi_{h}}{|b_{0}|+\frac{\alpha }{1-\beta }+\beta^{n}(|B_{0}|-\frac{\alpha }{1-\beta })}\;. \end{array}$$

Since *β* < 1, the dependence of *ψ*_*n*_ on *ξ*_*h*_ exponentially increases with *h*, because of the factor *β*^*n*−*h*^, and so the main contribution is given by the most recent extractions. We refer to this phenomenon as “local” reinforcement. The case *β* = 0 is an extreme case, for which *ψ*_*n*_ depends only on the last extraction *ξ*_*n*_. Note that, when *β* = 1, i.e. the case of the standard Pólya urn, all the past observations *ξ*_*h*_ equally contribute to *ψ*_*n*_, with a weight equal to *α*. This different dependence on the past leads to a different behaviour of *ψ*_*n*_ along time-steps (see [48]): in the standard Pólya urn, the process (*ψ*_*n*_) asymptotically stabilizes, converging almost surely toward a random variable, while in the RP urn, the process (*ψ*_*n*_) persistently fluctuates (see Figs 1–6).

If we set

$$\begin{array}{c} p_{0}=p_{0\;1}=\frac{b_{0\;1}}{|b_{0}|},\;\left(1-\gamma^{*}\right)=\frac{|b_{0}|}{r^{*}},\;{\tilde{B}}_{n}=\frac{B_{n\;1}}{|B_{n}|}\;, \end{array}$$

we get for a large *n*

$$\begin{array}{c} \begin{array}{cc} \psi_{n+1} & =\frac{b_{0\;1}}{r_{n+1}^{*}}+\frac{B_{n+1\;1}}{r_{n+1}^{*}}=\frac{|b_{0}|}{r_{n+1}^{*}}p_{0}+\frac{|B_{n+1}|}{r_{n+1}^{*}}{\tilde{B}}_{n+1}\\ & =\frac{|b_{0}|}{r_{n+1}^{*}}p_{0}+\frac{r_{n+1}^{*}-\left|b_{0}\right|}{r_{n+1}^{*}}{\tilde{B}}_{n+1}\\ & ∼\frac{|b_{0}|}{r^{*}}p_{0}+\frac{r^{*}-\left|b_{0}\right|}{r^{*}}{\tilde{B}}_{n+1}=\left(1-\gamma^{*}\right)p_{0}+\gamma^{*}{\tilde{B}}_{n+1} \end{array} \end{array}$$

and

$$\begin{array}{c} \begin{array}{cc} {\tilde{B}}_{n+1} & =\frac{B_{n+1}}{|B_{n+1}|}=\frac{\beta }{|B_{n+1}|}B_{n}+\frac{\alpha }{|B_{n+1}|}\xi_{n+1}\\ & =\beta \frac{r_{n}^{*}-\left|b_{0}\right|}{r_{n+1}^{*}-\left|b_{0}\right|}{\tilde{B}}_{n}+\frac{\alpha }{r_{n+1}^{*}-\left|b_{0}\right|}\xi_{n+1}\\ & ∼\beta {\tilde{B}}_{n}+\frac{\alpha }{r^{*}-\left|b_{0}\right|}\xi_{n+1}=\beta {\tilde{B}}_{n}+\left(1-\beta \right)\xi_{n+1}\;. \end{array} \end{array}$$

Summing up, the model dynamics can be approximated for *n* large by

$$\begin{array}{c} \psi_{n+1}=\left(1-\gamma^{*}\right)p_{0}+\gamma^{*}{\tilde{B}}_{n+1},\;{\tilde{B}}_{n+1}=\beta {\tilde{B}}_{n}+\left(1-\beta \right)\xi_{n+1}, \end{array}$$

where $p_{0},\;\gamma^{*},\;\beta ,\;{\tilde{B}}_{0}$ are the parameters. Note that *α* does not appear among the parameters for the above approximated dynamics, but it is included in the new parameter γ*. Moreover, the quantity ${\tilde{B}}_{0}$ is exponentially fast negligible, because we have ${\tilde{B}}_{n}=\beta^{n}{\tilde{B}}_{0}+\left(1-\beta \right)\sum_{h=1}^{n}\beta^{n-h}\xi_{h}$, with *β* < 1. Therefore, the fundamental parameters are *p*_0_, γ* and *β*: *p*_0_ is a deterministic component, γ* tunes the weight in the predictive mean *ψ*_*n*+1_ of the random “fluctuation” component ${\tilde{B}}_{n+1}$ with respect to the deterministic one, and *β* regulates the dependence of the present state ${\tilde{B}}_{n+1}$ on the previous state ${\tilde{B}}_{n}$ and on the present observation *ξ*_*n*+1_. We refer to ${\left({\tilde{B}}_{n}\right)}_{n}$ as the “fashion” process, since it reproduces the trend variations of the considered phenomenon (in our case, the sentiment of Twitter posts). In the following applications, we consider the following cases:

- *Complete RP model*: The three parameters *θ* = (*p*_0_, γ*, *β*) are free to vary.
- *“Only Fashion” RP model*: γ* = 1 (and *p*_0_ = 0 irrelevant). This means that the predictive mean is not driven by any deterministic component, but it coincides with the fashion process. The free parameter is given by *θ* = *β*.
- *“No Fashion” RP model*: γ* = 0 (and *β* = 0 irrelevant). In this case *ψ*_*n*_ is equal to the constant *p*_0_ and, consequently, the free parameter is given by *θ* = *p*_0_.

### 2.3 Model with delay

In applications, the extractions from the urn typically correspond to actions performed by agents. Therefore, it is plausible that there is a delay in information, in the sense that, when the agent decides to make the action that will appear at time-step *n* + 1, he/she only knows what happened until a certain time-step *t*(*n*)<*n*, i.e. the actions at time-steps 1, …, *t*(*n*). For instance, in our framework, the actions are the tweets and so it is plausible that, when the author of the tweet posted at time-step *n* + 1 is writing, he /she only knows the previous tweets until a certain time-step *t*(*n*)<*n*. In other words, we can image that an agent, after reading the tweets posted until time-step *t*(*n*), starts to write his/her tweet and posts it at time-step *n* + 1. Therefore, tweet *n* + 1 is not affected by tweets posted at time-steps *t*(*n*) + 1, …, *n*. When this is the case, the predictive means for action *n* + 1 are given by the composition of the urn until time-step *t*(*n*). In particular, if the number of colors is *c* = 2 and we denote by $I_{n}$ the information the agent has when performing action *n* + 1 (formally, $I_{0}$ equal to the trivial *σ*-field and $I_{n}=\sigma \left(\xi_{1},\ldots ,\xi_{t(n)}\right)$), we have

$$\begin{array}{c} {\hat{\psi }}_{n}=E\left[\xi_{n+1}|I_{n}\right]=P\left(\xi_{n+1}=1|I_{n}\right)=\frac{N_{t(n)\;1}}{N_{t(n)\;1}+N_{t(n)\;2}}=\psi_{t(n)}\;. \end{array}$$ (5)

Assuming to know the real time at which actions appeared (i.e., in our framework, the real time at which the posts are posted), a possible way to define *t*(*n*) is the following. Fix a value *D* > 0, divide (real) time in blocks of length *D* (choose *D* so that the blocks contain at least one action), define *j*(*n* + 1) the index of the time block containing the action *n* + 1 and set

$$\begin{array}{c} t\left(n\right)=\max\{t\in N:\;j\left(t\right)\leq {\left(j\left(n+1\right)-2\right)}_{+}\;\}\;. \end{array}$$

It follows that, for all actions appeared in a certain time block *j*, the missing information are the actions appeared in the immediately previous time block (i.e. block *j* − 1) plus the preceding actions of the same block. As a consequence, the quantity *D* is a lower bound for the delay and 2*D* an upper bound: agents looses at least *D* units of time and not more than 2*D* units of time.

In the following, we refer to this variant of the RP urn as “RP urn model with delay”.

## 3 Results

### 3.1 Data

Data have been collected from the Twitter platform, using the official API to Stream the exchange of messages on several topics. In the following, the various datasets are described in more details:

- **Italy, Migration debate**
  Data were collected through the Filter API since 23rd of January to 22nd of February 2019 and targeted the Italian debate on migration. Data were previously analysed in [16]. In the dataset, the information about the nature, automated or not (BOT or not), of the users is present. The BOT detection algorithm embedded is a lightweight version of the classifier proposed in [10]; more details on the dataset can be found in [16].
- **Italy, 10 days of traffic**
  The dataset collects the entire traffic, compatibly with the Filter API sampling, of messages in Italian in the days from the first to the 10th of September 2019: the keyword used for the query were the Italian vowels, in order to collect all messages that may contain some word. In the dataset, the information about the nature, automated or not (BOT or not), of the users is present. The BOT detection algorithm used was developed in [14].
- **Italy, COVID-19 epidemic**
  The dataset covers the period from February 21st to April to 20th 2020, including tweets in Italian language, and was previously analysed in [20]. The keywords used for the query are relative to the COVID-19 epidemic; more details can be found in the original reference. The dataset includes information on the automated or not (BOT or not) nature of the accounts, detected using the algorithm developed in [14].

For every message, the relative sentiment was calculated using the *polyglot* python module developed in [66], that provides a numerical value *v* ∈ [−1, 1] for the sentiment. We fix a threshold *T* = 0.35 so that we classify as a tweet with positive sentiment those with *v* > *T* and as a tweet with negative sentiment those with *v* < −*T*. We discard tweets with a value *v* ∈ [−*T*, *T*]. (There is not a particular reason for our choice of the value of *T*: indeed, we take the value 0.35 only because the interval [−1, 1] results divided into three parts of almost the same length. In Appendix, Sec. B, we show the results for other values of the threshold *T*).

Tables 1–3 show some descriptives of the samples obtained with *T* = 0.35:

**Table 1:** “Migration” sample: Descriptives of the sample obtained with *T* = 0.35.

Migration	Entire	Only BOTs’ posts
Posts	367367	4124
Percentage of positive posts	49.60%	47.97%

**Table 2:** “10 days of traffic” sample: Descriptives of the sample obtained with *T* = 0.35.

10 days of traffic	Entire	Only BOTs’ posts
Posts	3164620	102374
Percentage of positive posts	63.26%	63.79%

**Table 3:** “Covid” sample: Descriptives of the sample obtained with *T* = 0.35.

Covid	Entire	Only BOTs’ posts
Posts	2037584	48252
Percentage of positive posts	50.58%	54.00%

### 3.2 Analysis of the prediction ability

We apply the RP urn model with delay, ordering the tweets according to their creation time and taking each tweet with a positive/negative classification as an extraction in the urn model. More precisely, we apply the RP model with *c* = 2: the time series of the tweets represents the time series of the extractions from the urn, that is the random variables *ξ*_*n*_. The event {*ξ*_*n*_ = 1} means that tweet *n* exhibits a positive sentiment, while {*ξ*_*n*_ = 0} means that tweet *n* exhibits a negative sentiment. Moreover, we include a delay into the model as described in Subsec. 2.3, taking *D* = 3 minutes for the “migration” and “covid” samples and *D* = 30 seconds for the “10 days of traffic” sample. These values have been arbitrarily chosen, because we have no information about the real length of the delay. Notably, no significant difference has been observed taking zero delay.

The model parameters have been estimated by maximum likelihood. More precisely, we have divided the available *N* (ordered) actions (i.e. the tweets) into *S* slots of the same size (numbered from *s* = 0 to *s* = *S* − 1). For each slot *s* = 1, …, *S* − 1, with training data of the slots *s*′ = 0, …, *s* − 1, we have estimated the best parameters $\hat{\theta }\left(s\right)$ for the different proposed models (with delay): Standard Pólya, Complete RP, Only Fashion RP, No Fashion RP. (See Appendix, Sec. A, for a deeper exploration of the evolution of the parameters and further comments.) With these parameters, for each action *ξ*_*n*+1_ of the *s*-th slot, we have computed the conditional probability ${\hat{\psi }}_{n}$ (see Eq 5), that is the predictive mean for action *n* + 1, as a function of the estimated parameters and of the actions *ξ*_1_, …, *ξ*_*t*(*n*)_ (that is the information of the author of action *n* + 1). The predictive mean ${\hat{\psi }}_{n}$ represents our prediction of the future action *ξ*_*n*+1_. We have quantified the ability of the considered model to predict the future outcomes by means of the relative Squared Error (*SE*_*rel*_) with respect to the method that predicts the future outcome taking the value assumed by the majority in the past. More precisely, we have computed the following quantity:

$$\begin{array}{c} SE_{rel}=\frac{\sum_{n}{\left(\xi_{n+1}-m_{n}\right)}^{2}}{\sum_{n}{\left(\xi_{n+1}-{\hat{\psi }}_{n}\right)}^{2}}\;, \end{array}$$ (6)

where the sum is over all the observations except the ones in slot *s* = 0 and *m*_*n*_ is the value assumed by the majority of the past actions *ξ*_1_, …, *ξ*_*t*(*n*)_.

This quantity measures the ability of the model to predict the future outcomes: the greater it is, the better is the prediction with respect to the method based on the past majority. The values *SE*_*rel*_ obtained for the different considered models are also compared with the “theoretical” value of *SE*_*rel*_ computed replacing ${\hat{\psi }}_{n}$ by the mean value $\bar{\psi }$ on all the considered period. The term “theoretical” is used in order to point out that $\bar{\psi }$ is of course not a prediction, but it gives the *a posteriori* best constant fit once we have collected all the data until time-step *N*. Summing up, our aim is twofold: to obtain a value of *SE*_*rel*_ greater than 1, that means that the considered models beat the performance of the method based on the past majority, and to get a value greater or equal to the “theoretical” value, that means that the proposed models perform better or similarly than the (theoretical) *a posteriori* best constant fit.

We summarize the results in Tables 4–6. For each considered sample, we have also analysed the subset obtained by the restriction to the tweets classified as sent by a BOT. In the tables the best values are highlighted in bold. We can observe that, for the “Migration” and “covid” samples, the considered models perform more or less two times better than the method based on the past majority and this performance is similar to (indeed, in the most cases slightly better than) the one given by the (theoretical) *a posteriori* best constant fit. For the “10 days traffic” sample, the performance of the considered models is one and half times better than the method based on the past majority and this performance is similar to the one given by the (theoretical) *a posteriori* best constant fit. Moreover, the performances of the “Complete RP” model and of the “Only fashion RP” model do not significantly differ; while the “No Fashion RP” model performs less well. Therefore, in the next subsection, we will discard this last model.

**Table 4:** “Migration” sample (*T* = 0.35, *D* = 3′, *S* = 100 slots of equal size): Comparison of the different considered models in terms of (6).

Migration	Standard Pólya	Complete RP	Only Fashion RP	No Fashion RP	Theoretical value
Entire	200.91%	**206.28%**	206.22%	200.78%	200.93%
OnlyBOT	194.52%	199.63%	**199.75%**	194.24%	194.74%

**Table 5:** “10 days of traffic” sample (*T* = 0.35, *D* = 30′′, *S* = 100 slots of equal size): Comparison of the different considered models in terms of (6).

10 days of traffic	Standard Pólya	Complete RP	Only Fashion RP	No Fashion RP	Theoretical value
Entire	160.75%	**160.88%**	**160.88%**	160.74%	160.75%
OnlyBOT	159.57%	**159.70%**	159.62%	159.57%	159.58%

**Table 6:** “Covid” sample (*T* = 0.35, *D* = 3′, *S* = 1000 slots of equal size): Comparison of the different considered models in terms of (6).

Covid	Standard Pólya	Complete RP	Only Fashion RP	No Fashion RP	Theoretical value
Entire	199.97%	**203.15%**	**203.15%**	199.96%	200.02%
OnlyBOT	187.60%	190.43%	**190.47%**	187.58%	187.85%

(In Appendix, Sec. B, we collect results obtained with different thresholds *T* (used for the construction of the sample) and taking the slots (used for the parameters estimation) equal to the available days of observation).

### 3.3 Fluctuations of the sentiment curve

We provide some tables and figures in order to point out how the different considered models are able to reproduce the trend fluctuation of the sentiment curve. More precisely, in Figs 1–6, the yellow line is the cubic spline smoothing (Penalized Cubic regression splines with different numbers of nodes: *k* = 3, 5, 10, 20, 30, 50. See [67]) of the time series of the observed tweets {*ξ*_*n*_: *n* = 1, …, *N*}, together with the default confidence interval (gray), the red line represents the cubic spline smoothing (with the same number of nodes) of the time series of the estimated predictive means ${\hat{\psi }}_{n}$ (see Subsec. 3.2), obtained with the complete RP model with delay, the black and the blue lines provide similar approximations obtained with the other models with delay: black = Only fashion RP model and blue = Standard Pólya model. In Tables 7–12, we compare the different models by means of the Mean Squared Error (MSE), i.e.

$$\begin{array}{c} MSE=\frac{\sum_{n}{\left(\xi_{n+1}-{\hat{\psi }}_{n}\right)}^{2}}{\#observations}\;, \end{array}$$ (7)

where the sum is over all the observations except the ones in the slot *s* = 0 and *ξ*_*n*+1_ and ${\hat{\psi }}_{n}$ refer to the values on the curves with a given smoothing.

**Table 7:** “Migration” (*T* = 0.35, entire, *D* = 3′, *S* = 100 slots of equal size): MSE for different levels of smoothing.

smoothing	Only Fashion RP	Complete RP	Standard Pólya
no smooth	2.44 × 10^−1^	**2.43** × **10**^**−1**^	2.50 × 10^−1^
k = 3	**3.44** × **10**^**−9**^	1.41 × 10^−6^	3.03 × 10^−4^
k = 5	**1.19** × **10**^**−8**^	3.23 × 10^−6^	3.43 × 10^−4^
k = 10	**2.64** × **10**^**−7**^	1.74 × 10^−5^	1.64 × 10^−3^
k = 20	**1.04** × **10**^**−6**^	2.98 × 10^−5^	2.73 × 10^−3^
k = 30	**2.79** × **10**^**−6**^	4.03 × 10^−5^	3.83 × 10^−3^
k = 50	**7.18** × **10**^**−6**^	5.41 × 10^−5^	4.85 × 10^−3^

**Table 8:** “Migration” (*T* = 0.35, only BOTs’ posts, *D* = 3′, *S* = 100 slots of equal size): MSE for different levels of smoothing.

smoothing	Only Fashion RP	Complete RP	Standard Pólya
no smooth	2.43 × 10^−1^	**2.41** × **10**^**−1**^	2.50 × 10^−1^
k = 3	**2.62** × **10**^**−6**^	3.56 × 10^−5^	7.66 × 10^−4^
k = 5	4.19 × 10^−4^	**1.90** × **10**^**−4**^	1.10 × 10^−3^
k = 10	**1.03** × **10**^**−4**^	3.50 × 10^−4^	3.36 × 10^−3^
k = 20	**5.36** × **10**^**−4**^	8.19 × 10^−4^	6.58 × 10^−3^
k = 30	**9.09** × **10**^**−4**^	1.22 × 10^−3^	9.13 × 10^−3^
k = 50	2.53 × 10^−3^	**2.36** × **10**^**−3**^	1.34 × 10^−2^

**Table 9:** “10 days of traffic” (*T* = 0.35, entire, *D* = 30′′, *S* = 100 slots of equal size): MSE for different levels of smoothing.

smoothing	Only Fashion RP	Complete RP	Standard Pólya
no smooth	2.32 × 10^−1^	2.32 × 10^−1^	2.33 × 10^−1^
k = 3	**3.15** × **10**^**−9**^	2.61 × 10^−7^	1.22 × 10^−5^
k = 5	**3.86** × **10**^**−9**^	8.09 × 10^−7^	3.34 × 10^−5^
k = 10	**1.94** × **10**^**−8**^	2.02 × 10^−6^	6.88 × 10^−5^
k = 20	**7.81** × **10**^**−8**^	2.65 × 10^−6^	8.80 × 10^−5^
k = 30	**1.74** × **10**^**−7**^	2.86 × 10^−6^	9.65 × 10^−5^
k = 50	**1.08** × **10**^**−6**^	5.15 × 10^−6^	1.53 × 10^−4^

**Table 10:** “10 days traffic” (*T* = 0.35, only BOTs’ posts, *D* = 30′′, *S* = 100 slots of equal size): MSE for different levels of smoothing.

smoothing	Only Fashion RP	Complete RP	Standard Pólya
no smooth	2.31 × 10^−1^	2.31 × 10^−1^	2.31 × 10^−1^
k = 3	**4.10** × **10**^**−7**^	6.67 × 10^−7^	5.73 × 10^−6^
k = 5	**7.95** × **10**^**−7**^	2.02 × 10^−5^	5.97 × 10^−5^
k = 10	**6.81** × **10**^**−6**^	2.35 × 10^−5^	7.19 × 10^−5^
k = 20	**1.59** × **10**^**−5**^	5.43 × 10^−5^	1.52 × 10^−4^
k = 30	**2.59** × **10**^**−5**^	5.98 × 10^−5^	1.75 × 10^−4^
k = 50	**9.80** × **10**^**−5**^	1.23 × 10^−4^	3.49 × 10^−4^

**Table 11:** “Covid” (*T* = 0.35, entire, *D* = 3′, *S* = 1000 slots of equal size): MSE for different levels of smoothing.

smoothing	Only Fashion RP	Complete RP	Standard Pólya
no smooth	2.46 × 10^−1^	2.46 × 10^−1^	2.50 × 10^−1^
k = 3	**3.98** × **10**^**−8**^	7.37 × 10^−6^	2.58 × 10^−3^
k = 5	**5.51** × **10**^**−8**^	7.53 × 10^−6^	2.64 × 10^−3^
k = 10	**1.54** × **10**^**−7**^	8.63 × 10^−6^	2.92 × 10^−3^
k = 20	**7.93** × **10**^**−7**^	9.37 × 10^−6^	3.10 × 10^−3^
k = 30	**1.06** × **10**^**−6**^	9.80 × 10^−6^	3.24 × 10^−3^
k = 50	**2.06** × **10**^**−6**^	1.10 × 10^−5^	3.46 × 10^−3^

**Table 12:** “Covid” (*T* = 0.35, only BOTs’ posts, *D* = 3′, *S* = 1000 slots of equal size): MSE for different levels of smoothing).

smoothing	Only Fashion RP	Complete RP	Standard Pólya
no smooth	2.45 × 10^−1^	2.45 × 10^−1^	2.49 × 10^−1^
k = 3	**3.23** × **10**^**−6**^	5.33 × 10^−5^	3.38 × 10^−3^
k = 5	**1.16** × **10**^**−5**^	5.16 × 10^−5^	3.38 × 10^−3^
k = 10	**2.84** × **10**^**−5**^	6.88 × 10^−5^	3.53 × 10^−3^
k = 20	**5.70** × **10**^**−5**^	9.78 × 10^−5^	3.80 × 10^−3^
k = 30	**1.67** × **10**^**−4**^	1.81 × 10^−4^	4.01 × 10^−3^
k = 50	3.05 × 10^−4^	**2.94** × **10**^**−4**^	4.38 × 10^−3^

We can observe that, as explained before in Section 2.2, the RP urn model is able to reproduce the fluctuations of the observed sentiment curve, while the standard Pólya urn model produces a curve that converges to a value.

(In Appendix, Sec. B, we collect results obtained with different thresholds *T* (used for the construction of the sample) and taking the slots (used for the parameters estimation described in Subsec. 3.2) equal to the available days of observation).

## 4 Discussion and conclusions

Online Social Networks (OSN) represent a perfect environment for the study of the emotional reaction to public events. It has been observed that the sentiment of a message may be a driver for the diffusion of a message in online social networks [3, 4, 6, 21]. Interestingly, Ref. [3] shows that, on different arguments, the *sensitivity*, i.e. the emotional reaction to the event, finds a sort of stability.

Leveraging on this feature of the online debate, we apply here a modification of the Pólya urn model, embedding a “local” reinforcement effect [48, 53], representing a sort of “fashion” contribution and capturing the persistence of a common sentiment. Similarly to the standard Pólya urn, the future outcome depends on the past history, but, differently from the original model, in the Rescaled Pólya urn, the influence of the recent outcomes has a greater impact on future extractions. This represents the “fashion” effect and its introduction properly captures the evolution of the sentiment of the online debate. We also include in the model a delay in information as described in Subsection 2.3, introducing the Rescaled Pólya urn with delay.

The results collected in Subsection 3.3 show that indeed the Rescaled Pólya model outperforms greatly the standard Pólya model. Moreover, as shown in Subsection 3.2, the RP urn model permits to have reliable predictions from past observations. As told before, the employed model incorporates itself a delay in information and, in addition, the model parameters are fitted using the data from all the previous slots. As it can be observed from the evolution of the model parameters, all of them converge smoothly to a fixed value. Estimating the parameters using only the closest slots, is going to be the target of near future research, together with the examination of different definitions of the delay included in the model.

Summarising, the present paper has essentially two targets: to propose a novel model for the reproduction and the prediction of the sentiment in the online debate and to examine and study the implications of the Rescaled Pólya urn (with delay). Building a simple and realistic model improves our understanding of the phenomenon: in the particular case, the presence of a local reinforcement, i.e. the “fashion” effect described above, shows how persistent is the emotional reaction to a public event.

It is worth to be mentioned that the application to Online Social Media is one of the possible applications of the proposed model: due to its abstractness and generality, it can be applied to any kind of phenomenon showing a local “fashion” behaviour.

The Rescaled Pólya model is defined for any number *c* of colors. Therefore it is also possible to take into account the previously discarded tweets, say the “neutral” ones, i.e. those with sentiment between −0.35 and 0.35 (more generally, between −*T* and *T*). However, it is out of the scope of the present study. We do not think that taking into account more colors could produce a different finding. Indeed, the additional analyses related to different thresholds show that the choice of the threshold does not modify the essence of the outputs.

## Appendix

### A Parameters evolution

**Fig 7:**
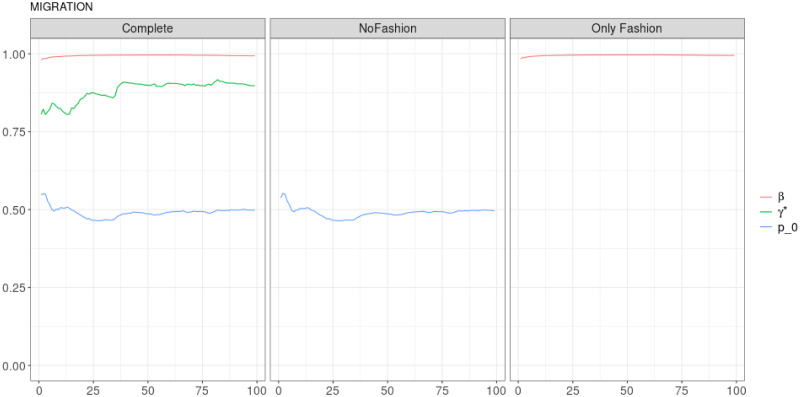
“Migration” (*T* = 0.35, entire, *D* = 3′): Model parameters evolution with *S* = 100 slots of equal size (i.e. 3673 observations). All parameters are slowly varying and converging to stable values.

**Fig 8:**
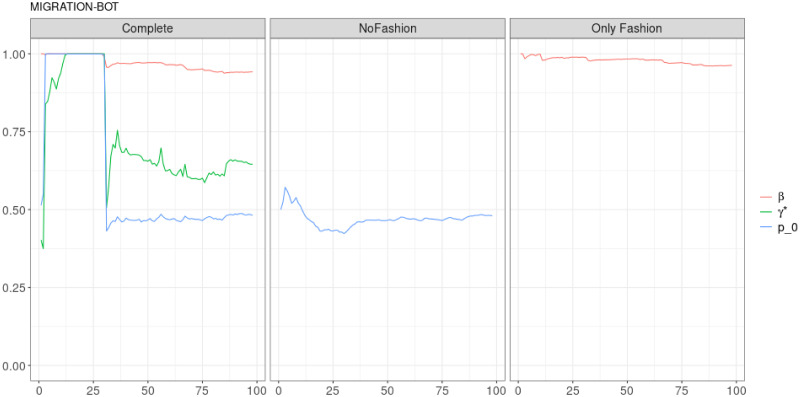
“Migration” (*T* = 0.35, only BOTs’ posts, *D* = 3′): Model parameters evolution with *S* = 100 slots of equal size (i.e. 41 observations). In the left panel, it is possible to distinguish an “Only fashion” initial phase (γ* ≃ 1) and more balanced phase (γ* ∈ [0.5, 0.75]).

As it is mentioned in the main text, in order to fit the parameters of the model, we divided the entire sample in slots of the same size (the number *S* of slots and their size for each considered sample is specified in the captions of the figures). Next, we use all slots previous to the one under consideration to fit the parameters. In this sense, we observe an evolution of the parameters as a matter of the evolution of the samples, which is different when focusing on the different nature of users in the debate. Such a difference is particularly evident in the Migration debate. Human accounts show a nearly constant parameter dynamics: while *β* is nearly constant in the Complete model, γ* and *p*_0_ display a smooth slow variation of nearly the 10% of their value, see Fig 7. The dynamics of the parameters for automated accounts is completely different, see Fig 8: in the Complete model, while *β* is slowly decreasing (but still experiencing a much greater decrease than the one observed for human accounts), parameters γ* and *p*_0_ display a step-like dynamics, ending shortly after *s* = 25.

**Fig 9:**
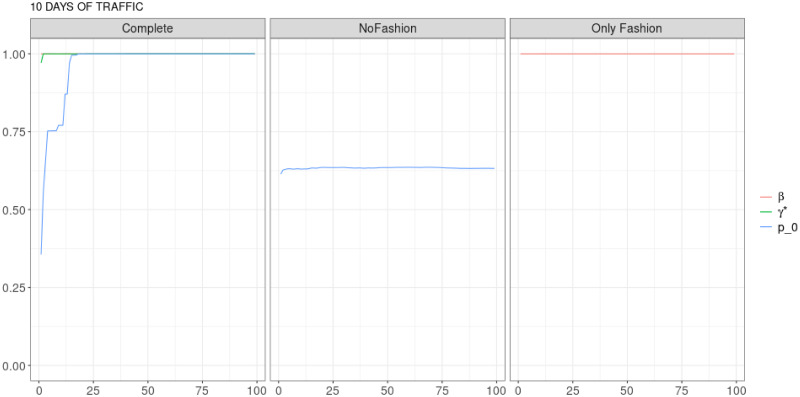
“10 days of traffic” (*T* = 0.35, entire, *D* = 30′′): Model parameters evolution with *S* = 100 slots of equal size (i.e. 31646 observations). All parameters for the complete model converge to 1 quite soon. With γ* ≃ 1, the Complete model is practically equivalent to the “Only Fashion” one.

**Fig 10:**
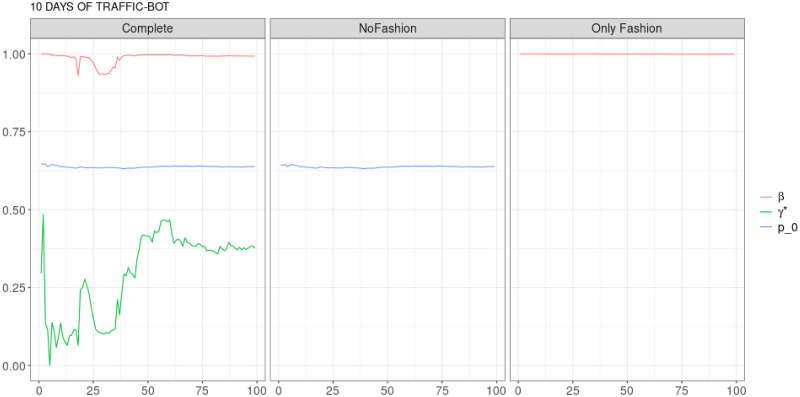
“10 days of traffic” (*T* = 0.35, only BOTs’ posts, *D* = 30′′): Model parameters evolution with *S* = 100 slots of equal size (i.e. 1023 observations). Every parameter of the complete model, but γ*, are essentially constant. Let us remark that γ* tunes the weight of the fashion process in the predictive mean.

A similar, but less evident, dynamics can be observed in the “10 days of traffic” sample, see Figs 9 and 10: in this case, all parameters converge to 1 quite soon in the case of the entire sample. Instead, we can see that *p*_0_ converge, but to something more than 0.6 quite immediately for the social bot subset, while the value of γ* oscillates between 0.5 and 0, before converging to something less than 0.4. The parameter *β* is nearly 1 for both cases.

**Fig 11:**
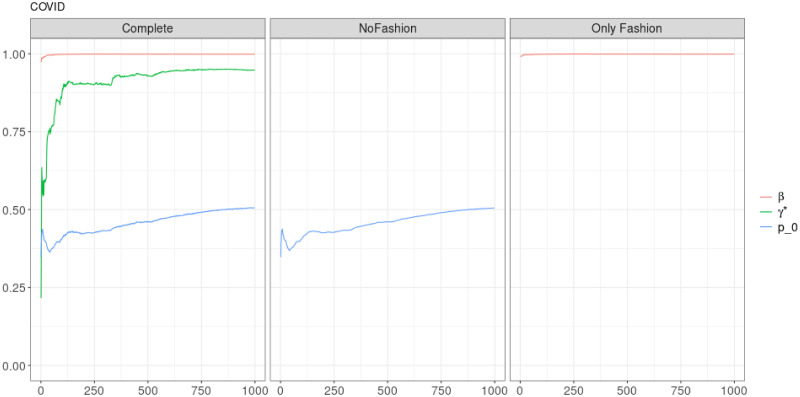
“Covid” (*T* = 0.35, entire, *D* = 3′): Model parameters evolution with *S* = 1000 slots of equal size (i.e. 2037 observations). All parameters are nearly constant or slowly converging.

**Fig 12:**
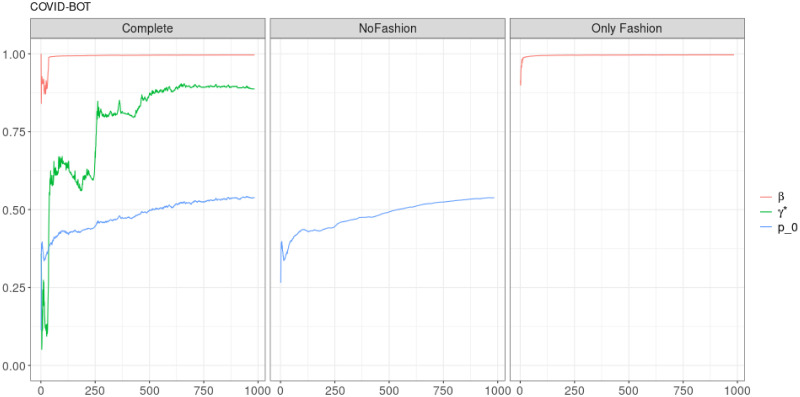
“Covid” (*T* = 0.35, only BOTs’ posts, *D* = 3′): Model parameters evolution with *S* = 1000 slots of equal size (i.e. 48 observations). Differently to the other samples, the parameters evolution for the Bots’ posts follows the one for the entire sample, displaying a greater noise contribution for γ*.

In the case of the online debate during the COVID-19 epidemic, Figs 11 and 12, the differences are extremely low, with the values of γ* quite flickering before converging to a value little lower than the one obtained for the entire sample. All other parameters are quite similar, both in the value and in the dynamics.

It is worthwhile to point out that, when we deal with the entire samples, the size of each slot is large enough in order to estimate properly the parameters from the very beginning. Therefore, we can guess that the behaviours of the curves in Figs 7, 9 and 11, before the stabilization, are really related to the dynamics of the debates. The situation is different when we deal only with posts sent by BOTs. Indeed, in this case, the initial estimation of the parameters may be affected by the limited size of the slots. However, also Figs 8, 10 and 12 exhibits a final stabilization of the values. An open question is if the differences in the values of the parameters and in their long-term dynamics observed when comparing the entire sample and the subset of BOTs’ posts is due to the different sizes or to an indeed different dynamics. This issue is not the focus of this work and it is going to be the target of further analyses.

### B Additional analyses

**Fig 13:**
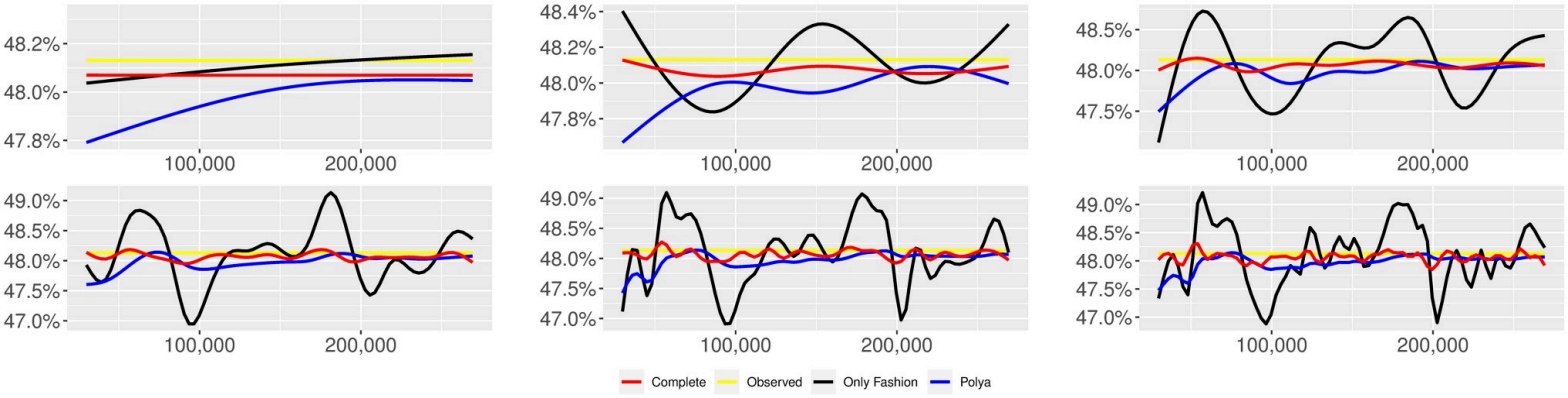
“Migration” (*T* = 0.5, entire, *D* = 3′, *S* = 100 slots of equal size): Sentiment curves. In each panel, the yellow line is the cubic spline smoothing of the time series of the observed tweets *ξ*_*n*+1_, together with the default confidence interval (gray), the red line represents the cubic spline smoothing of the time series of the estimated predictive means ${\hat{\psi }}_{n}$ (defined in Subsec. 3.2), obtained with the complete RP model, the black and the blue lines provide similar approximations obtained with the other models: black = Only fashion RP model and blue = Standard Pólya model. In each panel, the smoothing is obtained with a given number of nodes: *k* = 3 (top left panel), 5 (top middle panel), 10 (top right panel), 20 (bottom left panel), 30 (bottom middle panel), 50 (bottom right panel).

**Fig 14:**
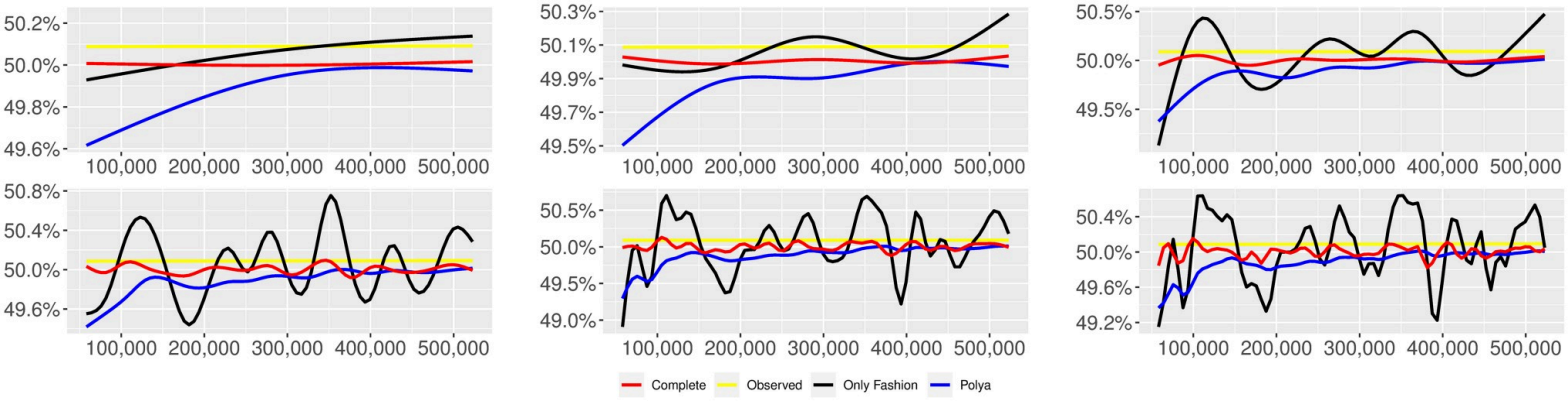
“Migration” (*T* = 0, entire, *D* = 3′, *S* = 100 slots of equal size): Sentiment curves. In each panel, the yellow line is the cubic spline smoothing of the time series of the observed tweets *ξ*_*n*+1_, together with the default confidence interval (gray), the red line represents the cubic spline smoothing of the time series of the estimated predictive means ${\hat{\psi }}_{n}$ (defined in Subsec. 3.2), obtained with the complete RP model, the black and the blue lines provide similar approximations obtained with the other models: black = Only fashion RP model and blue = Standard Pólya model. In each panel, the smoothing is obtained with a given number of nodes: *k* = 3 (top left panel), 5 (top middle panel), 10 (top right panel), 20 (bottom left panel), 30 (bottom middle panel), 50 (bottom right panel).

**Fig 15:**
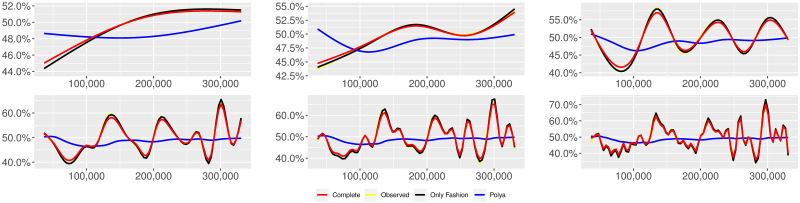
“Migration” (*T* = 0.35, entire, *D* = 3′, slots = days): Sentiment curves. In each panel, the yellow line is the cubic spline smoothing of the time series of the observed tweets *ξ*_*n*+1_, together with the default confidence interval (gray), the red line represents the cubic spline smoothing of the time series of the estimated predictive means ${\hat{\psi }}_{n}$ (defined in Subsec. 3.2), obtained with the complete RP model, the black and the blue lines provide similar approximations obtained with the other models: black = Only fashion RP model and blue = Standard Pólya model. In each panel, the smoothing is obtained with a given number of nodes: *k* = 3 (top left panel), 5 (top middle panel), 10 (top right panel), 20 (bottom left panel), 30 (bottom middle panel), 50 (bottom right panel).

**Fig 16:**
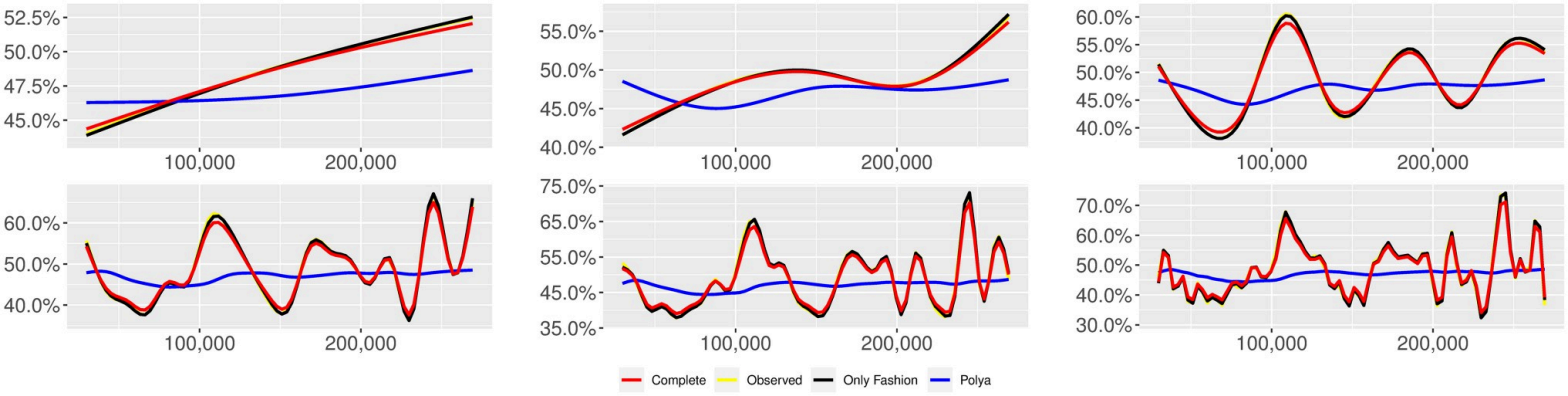
“Migration” (*T* = 0.5, entire, *D* = 3′, slots = days): Sentiment curves. In each panel, the yellow line is the cubic spline smoothing of the time series of the observed tweets *ξ*_*n*+1_, together with the default confidence interval (gray), the red line represents the cubic spline smoothing of the time series of the estimated predictive means ${\hat{\psi }}_{n}$ (defined in Subsec. 3.2), obtained with the complete RP model, the black and the blue lines provide similar approximations obtained with the other models: black = Only fashion RP model and blue = Standard Pólya model. In each panel, the smoothing is obtained with a given number of nodes: *k* = 3 (top left panel), 5 (top middle panel), 10 (top right panel), 20 (bottom left panel), 30 (bottom middle panel), 50 (bottom right panel).

**Fig 17:**
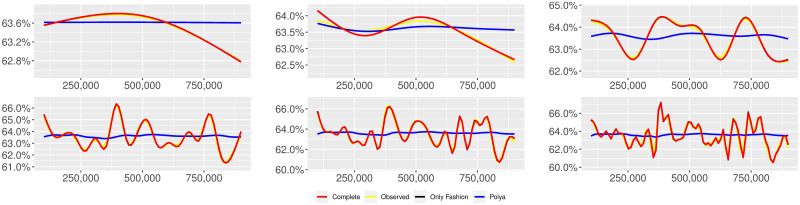
“10 days of traffic” (*T* = 0.5, entire, *D* = 30′′, *S* = 100 slots of equal size): Sentiment curves. In each panel, the yellow line is the cubic spline smoothing of the time series of the observed tweets *ξ*_*n*+1_, together with the default confidence interval (gray), the red line represents the cubic spline smoothing of the time series of the estimated predictive means ${\hat{\psi }}_{n}$ (defined in Subsec. 3.2), obtained with the complete RP model, the black and the blue lines provide similar approximations obtained with the other models: black = Only fashion RP model and blue = Standard Pólya model. In each panel, the smoothing is obtained with a given number of nodes: *k* = 3 (top left panel), 5 (top middle panel), 10 (top right panel), 20 (bottom left panel), 30 (bottom middle panel), 50 (bottom right panel).

**Fig 18:**
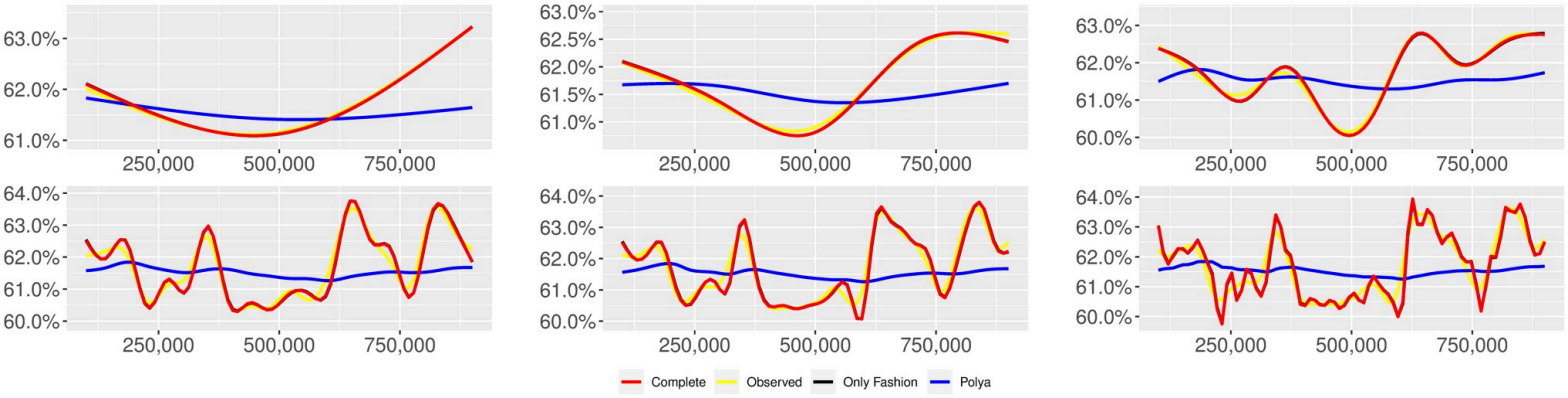
“10 days of traffic” (*T* = 0, entire, *D* = 30′′, *S* = 100 slots of equal size): Sentiment curves. In each panel, the yellow line is the cubic spline smoothing of the time series of the observed tweets *ξ*_*n*+1_, together with the default confidence interval (gray), the red line represents the cubic spline smoothing of the time series of the estimated predictive means ${\hat{\psi }}_{n}$ (defined in Subsec. 3.2), obtained with the complete RP model, the black and the blue lines provide similar approximations obtained with the other models: black = Only fashion RP model and blue = Standard Pólya model. In each panel, the smoothing is obtained with a given number of nodes: *k* = 3 (top left panel), 5 (top middle panel), 10 (top right panel), 20 (bottom left panel), 30 (bottom middle panel), 50 (bottom right panel).

**Fig 19:**
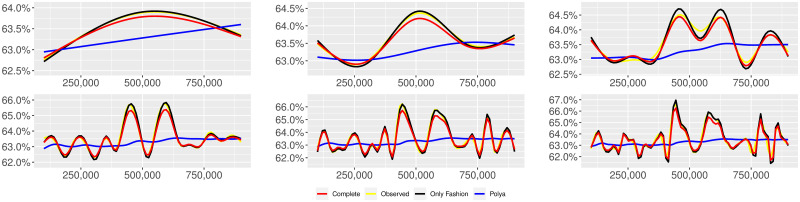
“10 days of traffic” (*T* = 0.35, entire, *D* = 30′′, slots = days): Sentiment curves. In each panel, the yellow line is the cubic spline smoothing of the time series of the observed tweets *ξ*_*n*+1_, together with the default confidence interval (gray), the red line represents the cubic spline smoothing of the time series of the estimated predictive means ${\hat{\psi }}_{n}$ (defined in Subsec. 3.2), obtained with the complete RP model, the black and the blue lines provide similar approximations obtained with the other models: black = Only fashion RP model and blue = Standard Pólya model. In each panel, the smoothing is obtained with a given number of nodes: *k* = 3 (top left panel), 5 (top middle panel), 10 (top right panel), 20 (bottom left panel), 30 (bottom middle panel), 50 (bottom right panel).

**Fig 20:**
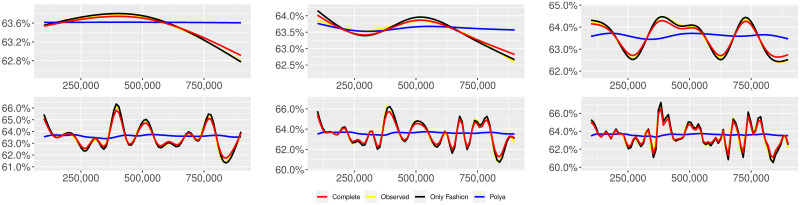
“10 days of traffic” (*T* = 0.5, entire, *D* = 30′′y, slots = days): Sentiment curves. In each panel, the yellow line is the cubic spline smoothing of the time series of the observed tweets *ξ*_*n*+1_, together with the default confidence interval (gray), the red line represents the cubic spline smoothing of the time series of the estimated predictive means ${\hat{\psi }}_{n}$ (defined in Subsec. 3.2), obtained with the complete RP model, the black and the blue lines provide similar approximations obtained with the other models: black = Only fashion RP model and blue = Standard Pólya model. In each panel, the smoothing is obtained with a given number of nodes: *k* = 3 (top left panel), 5 (top middle panel), 10 (top right panel), 20 (bottom left panel), 30 (bottom middle panel), 50 (bottom right panel).

**Fig 21:**
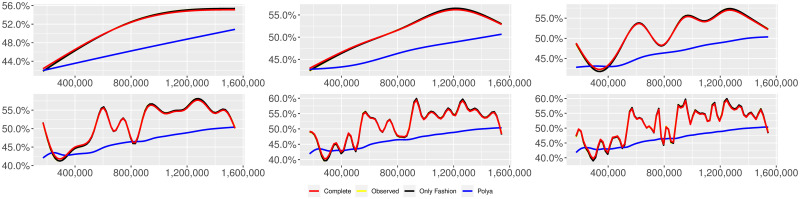
“Covid” (*T* = 0.5, entire, *D = 3′, *S* = 100 slots of equal size): Sentiment curves. In each panel, the yellow line is the cubic spline smoothing of the time series of the observed tweets *ξ*_*n*+1_, together with the default confidence interval (gray), the red line represents the cubic spline smoothing of the time series of the estimated predictive means ${\hat{\psi }}_{n}$ (defined in Subsec. 3.2), obtained with the complete RP model, the black and the blue lines provide similar approximations obtained with the other models: black = Only fashion RP model and blue = Standard Pólya model. In each panel, the smoothing is obtained with a given number of nodes: *k* = 3 (top left panel), 5 (top middle panel), 10 (top right panel), 20 (bottom left panel), 30 (bottom middle panel), 50 (bottom right panel).

**Fig 22:**
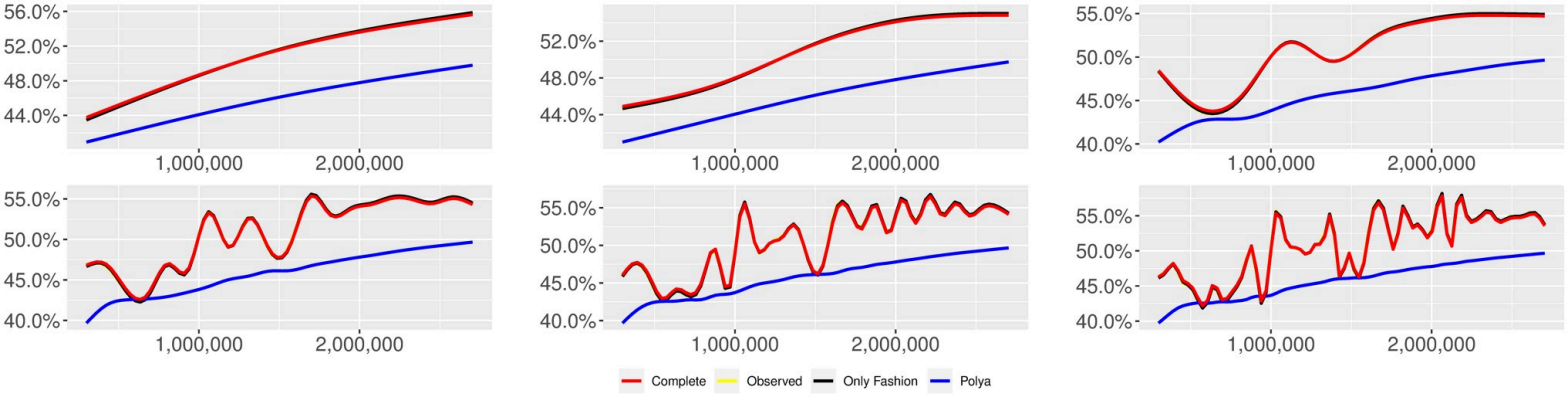
“Covid” (*T* = 0, entire, *D* = 3′, *S* = 100 slots of equal size): Sentiment curves. In each panel, the yellow line is the cubic spline smoothing of the time series of the observed tweets *ξ*_*n*+1_, together with the default confidence interval (gray), the red line represents the cubic spline smoothing of the time series of the estimated predictive means ${\hat{\psi }}_{n}$ (defined in Subsec. 3.2), obtained with the complete RP model, the black and the blue lines provide similar approximations obtained with the other models: black = Only fashion RP model and blue = Standard Pólya model. In each panel, the smoothing is obtained with a given number of nodes: *k* = 3 (top left panel), 5 (top middle panel), 10 (top right panel), 20 (bottom left panel), 30 (bottom middle panel), 50 (bottom right panel).

**Fig 23:**
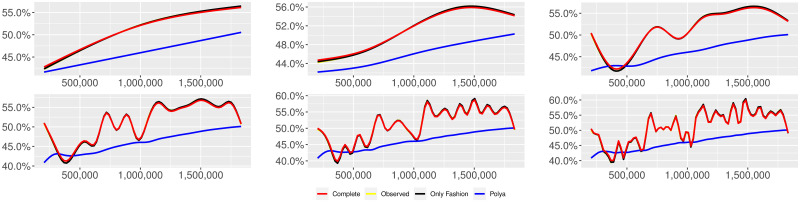
“Covid” (*T* = 0.35, entire, *D* = 3′, slots = days): Sentiment curves. In each panel, the yellow line is the cubic spline smoothing of the time series of the observed tweets *ξ*_*n*+1_, together with the default confidence interval (gray), the red line represents the cubic spline smoothing of the time series of the estimated predictive means ${\hat{\psi }}_{n}$ (defined in Subsec. 3.2), obtained with the complete RP model, the black and the blue lines provide similar approximations obtained with the other models: black = Only fashion RP model and blue = Standard Pólya model. In each panel, the smoothing is obtained with a given number of nodes: *k* = 3 (top left panel), 5 (top middle panel), 10 (top right panel), 20 (bottom left panel), 30 (bottom middle panel), 50 (bottom right panel).

**Fig 24:**
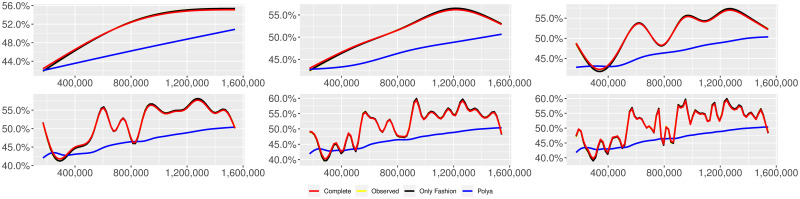
“Covid” (*T* = 0.5, entire, *D* = 3′, slots = days): Sentiment curves. In each panel, the yellow line is the cubic spline smoothing of the time series of the observed tweets *ξ*_*n*+1_, together with the default confidence interval (gray), the red line represents the cubic spline smoothing of the time series of the estimated predictive means ${\hat{\psi }}_{n}$ (defined in Subsec. 3.2), obtained with the complete RP model, the black and the blue lines provide similar approximations obtained with the other models: black = Only fashion RP model and blue = Standard Pólya model. In each panel, the smoothing is obtained with a given number of nodes: *k* = 3 (top left panel), 5 (top middle panel), 10 (top right panel), 20 (bottom left panel), 30 (bottom middle panel), 50 (bottom right panel).

We here collect the outputs of some additional analyses related to different choices of the threshold *T* for the construction of the sample and to a different partition into slots associated to the applied estimation technique for the model parameters. More precisely, we perform the analyses described in Subsecs. 3.2 and 3.3 for other two different thresholds (i.e. *T* = 0.5 and *T* = 0) and also taking the slots equal to the available days of observation. Table 13 reports the values of the indicator defined in Subsec. 3.2, Eq (6). Tables 14–25 and Figs 13–24 illustrate the results of the analyses described in Subsec. 3.3. We can observe that the main finding of this work does not change: indeed, since its local reinforcement mechanism, the RP urn model (with delay) is able to reproduce the fluctuations of the observed sentiment curve.

**Table 13:** Comparison of the different considered models in terms of (6).

Sample	Standard Pólya	Complete RP	Only Fashion RP	No Fashion RP	Theoretical value
Migration (T = 0, D = 3’, S = 100)	202.23%	**202.25%**	202.22%	202.22%	202.23%
Migration (T = 0.5, D = 3’, S = 100)	194.48%	**194.51%**	194.48%	194.48%	194.48%
Migration (T = 0.35, D = 3’, slots = days)	198.84%	203.11%	**203.46%**	198.02%	198.86%
Migration (T = 0.5, D = 3’, slots = days)	192.67%	198.40%	**198.78%**	191.10%	192.70%
10 days traffic (T = 0, D = 30”, S = 100)	165.68%	**165.79%**	**165.79%**	165.68%	165.68%
10 days traffic (T = 0.5, D = 30”, S = 100)	160.18%	**160.32%**	**160.32%**	160.17%	160.18%
10 days traffic (T = 0.35, D = 30”, slots = days)	158.10%	**158.23%**	**158.23%**	158.08%	158.10%
10 days traffic (T = 0.5, D = 30”, slots = days)	157.50%	**157.64%**	**157.64%**	157.48%	157.50%
Covid (T = 0, D = 3’, S = 100)	201.60%	**204.33%**	**204.33%**	201.51%	201.70%
Covid (T = 0.5, D = 3’, S = 100)	199.04%	202.24%	**202.25%**	198.91%	199.10%
Covid (T = 0.35, D = 3’, slots = days)	198.01%	201.08%	**201.11%**	197.82%	198.01%
Covid (T = 0.5, D = 3’, slots = days)	197.10%	200.20%	**200.23%**	196.85%	197.10%

**Table 14:** “Migration” (*T* = 0.5, entire, *D* = 3′, *S* = 100 slots of equal size): MSE for different levels of smoothing.

smoothing	Only Fashion RP	Complete RP	Standard Pólya
no smooth	2.50 × 10^−1^	2.50 × 10^−1^	2.50 × 10^−1^
k = 3	3.58 × 10^−7^	5.37 × 10^−7^	5.79 × 10^−6^
k = 5	4.13 × 10^−7^	5.36 × 10^−7^	8.22 × 10^−6^
k = 10	9.97 × 10^−6^	6.04 × 10^−7^	9.65 × 10^−6^
k = 20	1.10 × 10^−5^	2.11 × 10^−5^	2.91 × 10^−5^
k = 30	2.29 × 10^−5^	3.48 × 10^−5^	4.36 × 10^−5^
k = 50	3.44 × 10^−5^	4.51 × 10^−5^	5.73 × 10^−5^

**Table 15:** “Migration” (*T* = 0, entire, *D* = 3′, *S* = 100 slots of equal size): MSE for different levels of smoothing.

smoothing	Only Fashion RP	Complete RP	Standard Pólya
no smooth	2.50 × 10^−1^	2.50 × 10^−1^	2.50 × 10^−1^
k = 3	1.96 × 10^−8^	1.20 × 10^−6^	3.31 × 10^−6^
k = 5	1.77 × 10^−7^	9.52 × 10^−7^	4.03 × 10^−6^
k = 10	3.70 × 10^−6^	9.94 × 10^−7^	4.20 × 10^−6^
k = 20	4.29 × 10^−6^	7.86 × 10^−6^	1.12 × 10^−5^
k = 30	5.79 × 10^−6^	9.84 × 10^−6^	1.37 × 10^−5^
k = 50	1.26 × 10^−5^	1.79 × 10^−5^	2.36 × 10^−5^

**Table 16:** “Migration” (*T* = 0.35, entire, *D* = 3′, slots = days): MSE for different levels of smoothing.

smoothing	Only Fashion RP	Complete RP	Standard Pólya
no smooth	2.44 × 10^−1^	2.44 × 10^−1^	2.50 × 10^−1^
k = 3	7.26 × 10^−8^	1.76 × 10^−6^	3.01 × 10^−4^
k = 5	1.10 × 10^−7^	4.16 × 10^−6^	3.43 × 10^−4^
k = 10	1.24 × 10^−6^	2.84 × 10^−5^	1.64 × 10^−3^
k = 20	5.41 × 10^−6^	5.01 × 10^−5^	2.74 × 10^−3^
k = 30	1.20 × 10^−5^	6.92 × 10^−5^	3.84 × 10^−3^
k = 50	2.59 × 10^−5^	9.73 × 10^−5^	4.87 × 10^−3^

**Table 17:** “Migration” (*T* = 0.5, entire, *D* = 3′, slots = days): MSE for different levels of smoothing.

smoothing	Only Fashion RP	Complete RP	Standard Pólya
no smooth	2.42 × 10^−1^	2.42 × 10^−1^	2.50 × 10^−1^
k = 3	2.66 × 10^−7^	2.29 × 10^−6^	3.56 × 10^−4^
k = 5	6.47 × 10^−7^	2.49 × 10^−6^	3.75 × 10^−4^
k = 10	2.75 × 10^−6^	3.75 × 10^−5^	2.79 × 10^−3^
k = 20	6.37 × 10^−6^	5.80 × 10^−5^	3.92 × 10^−3^
k = 30	1.66 × 10^−5^	8.32 × 10^−5^	5.33 × 10^−3^
k = 50	3.15 × 10^−5^	1.09 × 10^−4^	6.54 × 10^−3^

**Table 18:** “10 days of traffic” (*T* = 0.5, entire, *D* = 30′′, *S* = 100 slots of equal size): MSE for different levels of smoothing.

smoothing	Only Fashion RP	Complete RP	Standard Pólya
no smooth	2.32 × 10^−1^	2.32 × 10^−1^	2.32 × 10^−1^
k = 3	4.32 × 10^−8^	4.49 × 10^−8^	7.88 × 10^−6^
k = 5	5.40 × 10^−9^	6.29 × 10^−9^	5.56 × 10^−5^
k = 10	1.43 × 10^−8^	1.70 × 10^−8^	7.56 × 10^−5^
k = 20	6.65 × 10^−8^	8.04 × 10^−8^	9.78 × 10^−5^
k = 30	2.62 × 10^−7^	2.89 × 10^−7^	1.04 × 10^−4^
k = 50	1.00 × 10^−6^	1.20 × 10^−6^	1.53 × 10^−4^

**Table 19:** “10 days of traffic” (*T* = 0, entire, *D* = 30′′, *S* = 100 slots of equal size): MSE for different levels of smoothing.

smoothing	Only Fashion RP	Complete RP	Standard Pólya
no smooth	2.37 × 10^−1^	2.37 × 10^−1^	2.37 × 10^−1^
k = 3	6.56 × 10^−9^	5.77 × 10^−9^	3.68 × 10^−5^
k = 5	7.12 × 10^−9^	5.97 × 10^−9^	5.20 × 10^−5^
k = 10	1.86 × 10^−8^	1.62 × 10^−8^	6.71 × 10^−5^
k = 20	7.66 × 10^−8^	6.46 × 10^−8^	7.92 × 10^−5^
k = 30	2.69 × 10^−7^	2.44 × 10^−7^	8.49 × 10^−5^
k = 50	1.23 × 10^−6^	1.02 × 10^−6^	1.25 × 10^−4^

**Table 20:** “10 days of traffic” (*T* = 0.35, entire, *D* = 30′′, slots = days): MSE for different levels of smoothing.

smoothing	Only Fashion RP	Complete RP	Standard Pólya
no smooth	2.32 × 10^−1^	2.32 × 10^−1^	2.33 × 10^−1^
k = 3	3.15 × 10^−9^	2.63 × 10^−7^	1.22 × 10^−5^
k = 5	3.86 × 10^−9^	8.15 × 10^−7^	3.34 × 10^−5^
k = 10	1.94 × 10^−8^	2.03 × 10^−6^	6.88 × 10^−5^
k = 20	7.81 × 10^−8^	2.68 × 10^−6^	8.80 × 10^−5^
k = 30	1.74 × 10^−7^	2.88 × 10^−6^	9.65 × 10^−5^
k = 50	1.08 × 10^−6^	5.19 × 10^−6^	1.53 × 10^−4^

**Table 21:** “10 days of traffic” (*T* = 0.5, entire, *D* = 30′′, slots = days): MSE for different levels of smoothing.

smoothing	Only Fashion RP	Complete RP	Standard Pólya
no smooth	2.32 × 10^−1^	2.32 × 10^−1^	2.32 × 10^−1^
k = 3	4.32 × 10^−8^	1.62 × 10^−7^	7.88 × 10^−6^
k = 5	5.40 × 10^−9^	1.94 × 10^−6^	5.56 × 10^−5^
k = 10	1.43 × 10^−8^	2.71 × 10^−6^	7.56 × 10^−5^
k = 20	6.65 × 10^−8^	3.65 × 10^−6^	9.78 × 10^−5^
k = 30	2.62 × 10^−7^	3.58 × 10^−6^	1.04 × 10^−4^
k = 50	1.00 × 10^−6^	5.86 × 10^−6^	1.53 × 10^−4^

**Table 22:** “Covid” (*T* = 0.5, entire, *D* = 3′, *S* = 100 slots of equal size): MSE for different levels of smoothing.

smoothing	Only Fashion RP	Complete RP	Standard Pólya
no smooth	2.46 × 10^−1^	2.46 × 10^−1^	2.50 × 10^−1^
k = 3	4.50 × 10^−8^	8.40 × 10^−6^	2.37 × 10^−3^
k = 5	5.77 × 10^−8^	8.51 × 10^−6^	2.42 × 10^−3^
k = 10	1.39 × 10^−7^	1.03 × 10^−5^	2.84 × 10^−3^
k = 20	1.14 × 10^−6^	1.19 × 10^−5^	3.12 × 10^−3^
k = 30	1.53 × 10^−6^	1.23 × 10^−5^	3.22 × 10^−3^
k = 50	2.84 × 10^−6^	1.39 × 10^−5^	3.49 × 10^−3^

**Table 23:** “Covid” (*T* = 0, entire, *D* = 3′, *S* = 100 slots of equal size): MSE for different levels of smoothing.

smoothing	Only Fashion RP	Complete RP	Standard Pólya
no smooth	2.47 × 10^−1^	2.47 × 10^−1^	2.50 × 10^−1^
k = 3	3.20 × 10^−8^	4.23 × 10^−6^	2.37 × 10^−3^
k = 5	4.57 × 10^−8^	4.32 × 10^−6^	2.40 × 10^−3^
k = 10	2.09 × 10^−7^	5.23 × 10^−6^	2.65 × 10^−3^
k = 20	7.14 × 10^−7^	5.56 × 10^−6^	2.72 × 10^−3^
k = 30	1.04 × 10^−6^	6.02 × 10^−6^	2.89 × 10^−3^
k = 50	1.70 × 10^−6^	6.91 × 10^−6^	3.06 × 10^−3^

**Table 24:** “Covid” (*T* = 0.35, entire, *D* = 3′, slots = days): MSE for different levels of smoothing.

smoothing	Only Fashion RP	Complete RP	Standard Pólya
no smooth	2.46 × 10^−1^	2.46 × 10^−1^	2.50 × 10^−1^
k = 3	3.98 × 10^−8^	7.43 × 10^−6^	2.58 × 10^−3^
k = 5	5.51 × 10^−8^	7.59 × 10^−6^	2.64 × 10^−3^
k = 10	1.54 × 10^−7^	8.70 × 10^−6^	2.92 × 10^−3^
k = 20	7.93 × 10^−7^	9.44 × 10^−6^	3.10 × 10^−3^
k = 30	1.06 × 10^−6^	9.88 × 10^−6^	3.24 × 10^−3^
k = 50	2.06 × 10^−6^	1.11 × 10^−5^	3.46 × 10^−3^

**Table 25:** “Covid” (*T* = 0.5, entire, *D* = 3′, slots = days): MSE for different levels of smoothing.

smoothing	Only Fashion RP	Complete RP	Standard Pólya
no smooth	2.46 × 10^−1^	2.46 × 10^−1^	2.50 × 10^−1^
k = 3	4.50 × 10^−8^	8.47 × 10^−6^	2.37 × 10^−3^
k = 5	5.77 × 10^−8^	8.58 × 10^−6^	2.42 × 10^−3^
k = 10	1.39 × 10^−7^	1.04 × 10^−5^	2.84 × 10^−3^
k = 20	1.14 × 10^−6^	1.20 × 10^−5^	3.12 × 10^−3^
k = 30	1.53 × 10^−6^	1.24 × 10^−5^	3.22 × 10^−3^
k = 50	2.84 × 10^−6^	1.40 × 10^−5^	3.49 × 10^−3^
